# Biologically Active Co(II) and Ni(II) Complexes of *N*-(2-Thienylmethylene)-2-Aminothiadiazole

**DOI:** 10.1155/MBD.2002.323

**Published:** 2002

**Authors:** Zahid H. Chohan

**Affiliations:** 1 Department of Chemistry Islamia University Bahawalpur Pakistan

## Abstract

Co(II) and Ni(II) complexes Schiff base, *N*-(2-thienylmethylene)-2-aminothiadiazole have been prepared and characterized by their physical, spectral and analytical data. The title Schiff-base acts as NNS donor tridentate during the complexation reaction with these metal ions having a composition, [M(L)_2_]X_n_ where M=Co(II) or Ni(II), L=, X=NO_3_^−^, SO_4_^2−^, C_2_O_4_^2−^ or CH_3_CO_2_^−^ and n=1 or 2 and show an octahedral geometry. In order to evaluate the effect anions upon chelation, the Schiff-base and its new complexes have been screened for their antibacterial activity against bacterial strains e.g., *Escherichia coli*, *Staphylococcus aureus*, and *Pseudomonas aeruginosa*.


### BIOLOGICALLY ACTIVE Co(II) AND Ni(II) COMPLEXES OF *N*-(2-THIENYLMETHYLENE)-2-AMINOTHIADIAZOLE

Zahid H. Chohan

Department of Chemistry, Islamia University, Bahawalpur, Pakistan

### ABSTRACT

Co(II) and Ni(II) complexes Schiff base, *N*-(2-thienylmethylene)-2-aminothiadiazole have been prepared and characterized by their physical, spectral and analytical data. The title Schiff-base acts as NNS donor tridentate during the complexation reaction with these metal ions having a composition, $[M(L)_2]X_n$ where M=Co(II) or Ni(II), L=, X=$NO_3^-$, $SO_4^{2-}$, $C_2O_4^{2-}$ or $CH_3CO_2^-$ and n=1 or 2 and show an octahedral geometry. In order to evaluate the effect anions upon chelation, the Schiff-base and its new complexes have been screened for their antibacterial activity against bacterial strains e.g., *Escherichia coli*, *Staphylococcus aureus*, and *Pseudomonas aeruginosa*.

### INTRODUCTION

Thiadiazole as ligand exhibits[1-3] interesting spectral and biological properties because of its strong heteroaromatic character. The azomethine linkage, aided by the adjacent donor heteroatoms act as a versatile function to make the molecule a useful participant in potentially important complexation reactions. Different studies[4-6] have indicated a strong relationship between the metal ions and/or their complexes with the potential ligands as promising antitumour[7-9] and antibacterial[10,11] agents. Many *in vivo* results have shown that compounds become more carcinostatic and bacteriostatic upon chelation[12]. These considerations, in continuation of the earlier work[13] done in this laboratory on the preparation of biologically active transition metal compounds inculcated more interest to further extend this area of research. Therefore, some more biologically active new metal complexes of the type $[M(L)_2]X_n$ where M=Co(II) or Ni(II), L=*N*-(2-thienylmethylene)-2-aminothiadiazole, X= $NO_3^-$, $SO_4^{2-}$, $C_2O_4^{2-}$ or $CH_3CO_2^-$ and n=1 or 2, having the same metal ion (cation) but, different anions are reported in this paper. The title Schiff-base along with its other analogues has already been reported[14]. Interesting biological properties of this NNS donor Schiff-base ligand compelled to prepare and report in the preceding paper these new metal complexes which describe the participating biological role of anions against bacterial strains e.g., *Escherichia coli*, *Staphylococcus aureus*, and *Pseudomonas aeruginosa*.

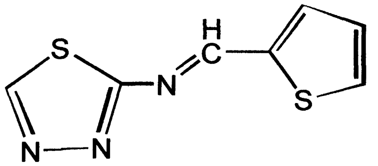

Fig 1. Structure of the Schiff base (L)

### EXPERIMENTAL

### Material and Methods

All chemicals and solvents used were of Analar grade. All metal(II) salts were used as chlorides. IR spectra were recorded on a Philips Analytical PU 9800 FTIR spectrophotometer. UV-Visible spectra were obtained in DMF on a Hitachi U-2000 double-beam spectrophotometer. C, H and N analyses was carried out by Butterworth Laboratories Ltd. Conductance of the metal complexes was determined in DMF on a Hitachi YSI-32 model conductometer. Magnetic measurements were made on solid complexes using the Gouy method. Melting points were recorded on a Gallenkamp apparatus and are uncorrected.

### Preparation of *N*-(2-thienylmethylene)-2-aminothiadiazole (L)

It was prepared[14] and characterized by the same method as reported earlier.

### Preparation of the Metal(II) Complexes.

A warm ethanol solution (20 mL) of the respective Schiff base (0.002 M) was added to a magnetically stirred solution of the metal(II) salt (0.001 M) in ethanol (25 mL). The mixture was refluxed for 1 h and cooled to room temperature. On cooling, precipitates were formed which were filtered, washed with ethanol, acetone and ether, and dried. Crystallization in aqueous ethanol (30:70) gave the desired metal complex. All other metal complexes were prepared respectively following the same method.

**Antibacterial Studies**

The synthesized metal complexes, in comparison to the uncomplexed Schiff-base ligands were screened for their antibacterial activity against pathogenic bacterial species, *Escherichia coli, Staphylococcus aureus* and *Pseudomonas aeruginosa*. The paper disc diffusion method[13] was adopted for the determination of antibacterial activity.

### RESULTS AND DISCUSSION

**Physical Properties**

The Schiff-base (L) (Fig. 1) was prepared by refluxing an appropriate amount of 2-amino-1,3,4-thiadiazole and thiophene-2-carboxaldehyde in ethanol in a 1:1 molar ratio. This Schiff-base was further used for complexation with the Co(II) and Ni(II) metal ions. All of the newly synthesized metal complexes (Table 1) were prepared by the stoichiometric reaction of the respective metals as their nitrate, sulfate, acetate and oxalate salts and the corresponding Schiff-base in a molar ratio M:L of 1:2 (Scheme 1). These complexes are air and moisture stable, intensely colored, amorphous solids which decompose above 200° C. They are insoluble in common organic solvents like ethanol, methanol, chloroform or acetone but soluble in DMSO and DMF. The molar conductances of the complexes dissolved in DMF fall into the range 143-147 $ohm^{-1}cm^2mol^{-1}$) indicating[15] that they are all electrolytes.

$$MX_n + 2L \longrightarrow [M(L)_2](X)_n$$

M=Co(II) or Ni(II)

L=Fig.1

$X=SO_4^{2-}, NO_3^-, C_2O_4^{2-}$ or $CH_3CO_2^-$

n=1 or 2

(Scheme 1)

Table 1. Physical and Analytical Data of the Metal(II) Complexes

| No | Metal chelate/ Mol. Formula | | Yield (%) | M.P (°C) (decomp) | B.M. ($\mu_{eff}$) | Calc (Found)% C H N |
|---|---|---|---|---|---|---|
| (1) | $[Co(L)_2](SO_4)$ $C_{14}H_{10}CoN_6O_5S_4$ | [528.9] | 62 | 206-208 | 4.5 | 31.8 1.9 15.9 (32.0) (1.7) (15.5) |
| (2) | $[Co(L)_2](NO_3)_2$ $C_{14}H_{10}CoN_8O_7S_3$ | [556.9] | 60 | 210-212 | 4.6 | 30.2 .1.8 20.1 (30.5) (2.0) (20.2) |
| (3) | $[Co(L)_2](C_2O_4)$ $C_{16}H_{10}CoN_6O_5S_3$ | [520.9] | 59 | 209-211 | 4.7 | 36.9 1.9 16.1 (36.6) (1.7) (16.6) |
| (4) | $[Co(L)_2](CH_3CO_2)_2$ $C_{18}H_{16}CoN_6O_5S_3$ | [550.9] | 61 | 213-215 | 4.6 | 39.2 2.9 15.2 (39.6) (3.0) (15.1) |
| (5) | $[Ni(L)_2](SO_4)$ $C_{14}H_{10}NiN_6O_5S_4$ | [528.7] | 60 | 215-217 | 3.3 | 31.7 1.9 15.9 (31.5) (1.4) (16.2) |
| (6) | $[Ni(L)_2](NO_3)_2]$ $C_{14}H_{10}NiN_6O_5S_4$ | [556.7] | 61 | 212-214 | 3.2 | 30.1 1.8 20.1 (30.5) (1.6) (20.3) |
| (7) | $[Ni(L)_2](C_2O_4)$ $C_{14}H_{10}NiN_6O_5S_4$ | [520.7] | 59 | 211-213 | 3.4 | 36.9 1.9 16.1 (36.8) (2.2) (16.5) |
| (8) | $[Ni(L)_2](CH_3CO_2)_2$ $C_{14}H_{10}NiN_6O_5S_4$ | [550.7] | 61 | 216-218 | 3.3 | 39.2 2.9 15.2 (39.5) (2.8) (15.0) |

**Infrared Spectra**

The IR spectra of the Schiff-base indicated that stretching vibrations due to the carbonyl ν(C=O) and ν($NH_2$) functions found at 1735 and 3420 $cm^{-1}$ disappeared in the spectra of its metal complexes and, instead, a strong new band appeared at ~1625 $cm^{-1}$ assigned[16] to the azomethine ν(HC=N) linkage. It however, suggested that the amino and aldehyde moieties of the starting reagents no more existed and converted into the Schiff-base compound showing, in turn, the azomethine linkage ν(HC=N) (Fig.1). The comparison of the IR spectra of the Schiff-base and its metal chelates (Table 2) further indicated that the Schiff-base was coordinated to the metal atom from mainly three donor sites hence, acting in a tridentate manner. The band originally appearing at 1625 $cm^{-1}$ due to the azomethine shifted to lower frequency by ~10-15 $cm^{-1}$ suggesting[17] participation of the azomethine nitrogen in complexation. A band at 1610 $cm^{-1}$ assigned to thiadiazole ring ν(C=N) nitrogen also shifted to lower frequency by ~10-15 $cm^{-1}$ that was also indicative of the involvement of the ring nitrogen of thiadiazole in complexation. A further evidence of the coordination of the Schiff-base with the metal atom, was shown by the appearance of weak low frequency new bands at 530 and 645 $cm^1$ assigned[18] to the metal-nitrogen ν(M-N) and metal-sulfur ν(M-S) respectively. These new bands were observable only in the spectra of the metal complexes and not in its uncomplexed Schiff-base which confirmed the participation of the donor groups (sulfur of thiophene and nitrogen of thiadiazole moieties) in the coordination.

Table 2. IR and UV-Visible Spectral Data of the Metal(II) Complexes.

| No | IR ($cm^{-1}$) | $\lambda_{max}$ ($cm^{-1}$) |
|---|---|---|
| 1 | 1615 (s, HC=N), 1585 (s, C=N), 645 (ms, M-S), 530 (ms, M-N) | 30,115, 17,470, 8795 |
| 2 | 1610 (s, HC=N), 1580 (s, C=N), 645 (ms, M-S), 530 (ms, M-N) | 29,875, 17,385, 8725 |
| 3 | 1610 (s, HC=N), 1585 (s, C=N), 640 (ms, M-S), 530 (ms, M-N) | 29,995, 17,415, 8680 |
| 4 | 1610 (s, HC=N), 1580 (s, C=N), 640 (ms, M-S), 535 (ms, M-N) | 30,010, 17,450, 8665 |
| 5 | 1615 (s, HC=N), 1580 (s, C=N), 645 (ms, M-S), 630 (ms, M-N) | 29,215, 16,140, 10,235 |
| 6 | 1610 (s, HC=N), 1585 (s, C=N), 645 (ms, M-S), 535 (ms, M-N) | 29,185, 16,205, 10,175 |
| 7 | 1615 (s, HC=N), 1585 (s, C=N), 645 (ms, M-S), 535 (ms, M-N) | 29,245, 16,155, 10,190 |
| 8 | 1615 (s, HC=N), 1585 (s, C=N), 645 (ms, M-S), 535 (ms, M-N) | 29,265, 16,160, 10215 |

s=sharp, ms=medium sharp

**Magnetic moment and UV-Visible Spectra**

The nature of the ligand field around the metal ion and the geometry of the complexes have been deduced from the electronic spectra and magnetic moment data. The room temperature magnetic moment of the solid cobalt(II) complexes was found to lie in the range (4.5-4.7 B.M), indicative[19] of three unpaired electrons per Co(II) ion in an octahedral environment. The nickel(II) complexes showed $\mu_{eff}$ values (3.3-3.4 B.M), corresponding[20] to two unpaired electrons per Ni(II) ion for their ideal six-coordinated configuration.

The electronic spectra of the Co(II) complexes showed three bands observed at 8,665-8,795, 17,385-17,470 and 29,875-30,115 $cm^{-1}$ which may be assigned to $^4T_{1g} \rightarrow {}^4T_{2g}(F)$, $^4T_{1g} \rightarrow {}^3A_{2g}(F)$ and $^4T_{1g} \rightarrow {}^4T_{1g}(P)$ transitions respectively and are suggestive[21] of the octahedral geometry around the cobalt ions.

The Ni(II) complexes exhibited three spin-allowed bands at 10,175-10,235, 16,140-16,205, and 29,185-29,265 $cm^{-1}$ assignable[22] respectively, to the transitions $^3A_{2g}(F) \rightarrow {}^3T_{2g}(F)(\nu_1)$, $^3A_{2g}(F) \rightarrow {}^3T_{1g}(F)(\nu_2)$ and $^3A_{2g}(F) \rightarrow {}^3T_{2g}(P)(\nu_3)$ which were characteristic of their octahedral geometry.

On the basis of the above observations, it is tentatively suggested that Co(II) and Ni(II) complexes show an octahedral geometry in which the two Schiff-bases act as tridentate and possibly accommodate themselves around the metal atom in such a way that a stable chelate ring of the complex is formed hence, giving a stable structure to the complex (Fig 2).

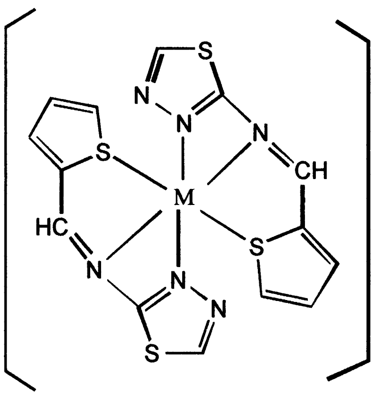

$X_n$

$X = NO_3^-$, $C_2O_4^{2-}$, $CH_3CO_2^-$ or $SO_4^{2-}$, n = 1 or 2

M = Co(II) or Ni(II)

Fig. 2: Proposed Structure of the Metal(II) Complexes (1-8).

**Antibacterial Properties**

The title Schiff-base and its Co(II) and Ni(II) metal chelates having the same metal cation but different anions, were evaluated for their antibacterial activity against bacterial species *Escherichia coli (a), Staphylococcus aureus (b) and Pseudomonas aeruginosa (c).* The compounds were tested at a concentration of 30 μg/0.01 mL in DMF solution using the paper disc diffusion method. The susceptibility zones were measured in diameter (mm) and the results are reproduced in Table 3. The susceptibility zones measured were the zones around the discs killing the active bacteria.

The Schiff base and its complexes individually exhibited varying degrees of inhibitory effects on the growth of the tested bacterial species. The antibacterial results evidently show that the activity of the Schiff base became more pronounced when coordinated to the metal ions. The metal ions having different anions had varying antibacterial influence on bacterial species. For example, the Co(II) complex with nitrate anion was more bactericidal than the Co(II) complex with sulfate, oxalate or acetate anions. Similarly, the Cu(II) complex oxalate was more antibacterial than the complex having acetate, chloride or sulfate. Similar results were found in the case of Ni(II) complexes. It was observed that the order of potency, in comparison to the metal complexes having chloride anions evaluated and reported[14] earlier, is as follows,

$$NO_3^- > C_2O_4^{2-} > CH_3CO_2^- > Cl^- > SO_4^{2-}$$

The results of antibacterial studies clearly show that the process of chelation dominantly affects the overall biological behavior of the compounds, which are potent against bacterial strains. The results of present studies however, indicate that different anions that stay outside the coordination sphere of the complex also play a significant role in this biological process. It is suspected that factors such as solubility or dipole moment observed by different anions may effect the cell permeability mechanisms through the lipoid layer of the organisms thus killing of them more effectively. Our *in vitro* studies are in progress, which may help us in establishing the exact biological role of these anions.

Table 3. Antibacterial Activity Data of the Schiff-base and its Metal Complexes

| Schiff base/ Complex | Microbial Species a | b | c |
|---|---|---|---|
| L | ++ | + | ++ |
| (1) | +++ | +++ | +++ |
| (2) | +++ | ++ | ++++ |
| (3) | +++ | +++ | +++ |
| (4) | ++++ | +++ | +++ |
| (5) | +++ | ++ | ++++ |
| (6) | +++ | +++ | +++ |
| (7) | ++++ | +++ | ++++ |
| (8) | ++++ | +++ | +++ |

a= *Escherichia coli*, b= *Staphylococcus aureus*,
c= *Pseudomonas aeruginosa*

Inhibition zone diameter mm (% inhibition): +, 6-10 (27-45 %); ++, 10-14 (45-64 %); +++, 14-18 (64-82 %); ++++, 18-22 (82-100 %). Percent inhibition values are relative to inhibition zone (22 mm) of the most active compound with 100 % inhibition.

### ACKNOWLEDGEMENT

The authors gratefully acknowledge the Department of Pathology, Qaid-e-Azam Medical College, Bahawalpur, Pakistan in undertaking the antibacterial studies.

### REFERENCES

1. G. Kornis, "1,3,4-Thiadiazoles", in Comprehensive Heterocyclic Chemistry", Pergamon Press, Vol. 6, (1984).
2. Z. H. Chohan, M. F. Jaffery and C. T. Supuran, Metal-Based Drugs, **8**, 95 (2001).
3. Z. H. Chohan, H. Pervez, A. Rauf and C. T. Supuran, Metal-Based Drugs, **8**, 263 (2002).
4. D. R. Williams, "*The Metals of Life*", Van Nostrand, London, (1971).
5. D. H. Brown, W. E. Smith, J. W. Teape and A. J. Lewis, *J. Med. Chem*, **23**, 729 (1980).
6. a) R. J. P. William, "*Quart. Rev*, **24**, 331 (1970); b) R. J. P. William, "*Bioinorganic Chemistry*", American Chemical Society, Washington, (1971).
7. B. Rosenberg and L. V. Camp, *Cancer. Res*, **30**, 1799 (1970)
8. M. J. Clare and J. D. Heeschele, *Bioinorg. Chem*, **2**, 187 (1973).
9. V. L. Narayanan, M. Nasr and K. D. Paull, in "*Tin-Based Antitumor Drugs*", M. Gielen (Ed), NATO ASI Series, Vol. H37, Springer Verlag, Berlin (1990).

10. A. J. Crowe, in "*Metal Based Antitumor Drugs*", M. Gielen (Ed), Vol 1, Freund, London (1988).
11. M. J. Seven and L. A. Johnson, "*Metal Binding in Medicine*", Lippincott Co, Philadelphia, PA, 4th Ed (1960).
12. V. K. Patel, A. M. Vasanwola and C. R. Jejurkas, *Ind. J. Chem*, **28A**, 719 (1989).
13. a) Z. H. Chohan and S. K. A. Sherazi, *Synth. React. Inorg. Met.-Org. Chem*, **29**, 105 (1999); b) Z. H. Chohan and S. Kausar, *Metal-Based Drugs*, 7, 17 (2000); c) Z. H. Chohan and M. Praveen, *Appl. Organometal. Chem*, **14**, 376 (2000) d) Z. H. Chohan and M. Praveen, *Synth. React. Inorg. Met.-Org. Chem*, **30**, 175 (2000); e) Z. H. Chohan and M. Praveen, *Appl. Organometal. Chem*, 15, 617 (2001); f) Z. H. Chohan, A. Munawar and C. T. Supuran, *Metal-Based Drugs*, **8**, 137 (2001); g) Z. H. Chohan, *Metal-Based Drugs*, 7, 177 (2000); h) Z. H. Chohan, M. A. Farooq and M. S. Iqbal, *Metal-Based Drugs*, 7, 133 (2000).
14. Zahid H. Chohan, Humayun Pervez, Abdul Rauf, Andrea Scozzafava, and Claudiu T. Supuran, *J. Enz. Inhib* (in press).
15. a) A. M. Shallary, M. M. Moustafa and M. M. Bekheit, *J. Inorg. Nucl. Chem*, **41**, 267 (1979). b) W. J. Geary, *Coord. Chem. Rev*, 7, 81 (1971).
16. L. J. Bellamy, "*The Infrared Spectra of Complex Molecules*", 3rd Ed, Methuen, London, (1966).
17. M. Yongxiang, Z. Zhengzhi, M. Yun and Z. Gang, *Inorg. Chim. Acta*, **165**, 185 (1989).
18. K. Nakamoto, "*Infrared Spectra of Inorganic and Coordination Compounds*", 2nd Ed, Wiley Interscience, New York, (1970).
19. C. J. Balhausen, "An Introduction to Ligand Fields", McGraw Hill, New York (1962).
20. a) B. N. Figgis and J. Lewis, *J. Prog. Inorg. Chem*, **6**, 197 (1967); b) A. B. P. Lever, Inorg. Chem, 4, 763 (1965); c) M. D. Glick and R. L. Lintvedt, *Prog. Inorg. Chem*, **21**, 233 (1976); d) R. S..Drago, "*Physical Methods in Inorganic Chemistry*", Reinhold, New York, (1965); e) B. N. Figgis, "*Introduction to Ligand Fields*", J. Wiley, New York, (1976).
21. a) B. P. Lever, "*Inorganic Electronic Spectroscopy*", Elsevier, Amsterdam, (1984); b) T. M. Dunn, J. Lewis and R. C. Wilkins, "*The Visible and Ultraviolet Spectra of Complex Compounds in Modern Coordination Chemistry*", Interscience, New York, (1960).
22. a) A. B. P. Lever, J. Lewis and R. S. Nyholm, *J. Chem. Soc*, 4761 (1964); b) R. L. Carlin,., "*Transition Metal Chemistry*", Ed, R. L. Carlin, Vol 1, Marcel Decker, New York, (1965)

**Received: April 16, 2002 – Accepted: May 8, 2002 –**
**Accepted in publishable format: May 9, 2002**

